# 5-ALA Fluorescence Is a Powerful Prognostic Marker during Surgery of Low-Grade Gliomas (WHO Grade II)—Experience at Two Specialized Centers

**DOI:** 10.3390/cancers13112540

**Published:** 2021-05-21

**Authors:** Arthur Hosmann, Matthias Millesi, Lisa I. Wadiura, Barbara Kiesel, Petra A. Mercea, Mario Mischkulnig, Martin Borkovec, Julia Furtner, Thomas Roetzer, Stefan Wolfsberger, Joanna J. Phillips, Anna S. Berghoff, Shawn Hervey-Jumper, Mitchel S. Berger, Georg Widhalm

**Affiliations:** 1 Department of Neurosurgery, Medical University of Vienna, 1090 Vienna, Austria; arthur.hosmann@meduniwien.ac.at (A.H.); matthias.millesi@meduniwien.ac.at (M.M.); lisa.wadiura@meduniwien.ac.at (L.I.W.); barbara.kiesel@meduniwien.ac.at (B.K.); petra.mercea@meduniwien.ac.at (P.A.M.); mario.mischkulnig@meduniwien.ac.at (M.M.); martin.borkovec@skyforge.at (M.B.); stefan.wolfsberger@meduniwien.ac.at (S.W.); 2 Comprehensive Cancer Center—Central Nervous System Tumours Unit (CCC-CNS), Medical University of Vienna, 1090 Vienna, Austria; Thomas.roetzer@meduniwien.ac.at (T.R.); anna.berghoff@meduniwien.ac.at (A.S.B.); 3 Division of Neuroradiology and Musculoskeletal Radiology, Department of Biomedical Imaging and Image-guided Therapy, Medical University of Vienna, 1090 Vienna, Austria; julia.furtner@meduniwien.ac.at; 4 Division of Neuropathology and Neurochemistry, Department of Neurology, Medical University of Vienna, 1090 Vienna, Austria; 5 Department of Pathology, University of California, San Francisco (UCSF), CA 94143, USA; joanna.phillips@ucsf.edu; 6 Division of Oncology, Department of Medicine I, Medical University of Vienna, 1090 Vienna, Austria; 7 Department of Neurological Surgery, University of California, San Francisco (UCSF), CA 94143, USA; Shawn.Hervey-Jumper@ucsf.edu (S.H.-J.); Mitchel.Berger@ucsf.edu (M.S.B.)

**Keywords:** 5-ALA, fluorescence, low-grade gliomas, patient prognosis

## Abstract

**Simple Summary:**

5-aminolevulinic acid (5-ALA) is administered orally before brain tumor surgery to improve intraoperative visualization of tumor tissue. In comparison to most of the aggressive high-grade gliomas, only a few low-grade gliomas (LGG) demonstrate visible fluorescence during surgery. As the prognosis of these LGG is hard to predict, we aimed to investigate if visible fluorescence might be an intraoperative marker for aggressive tumor behavior in patients with LGG. According to our data, we could demonstrate that intraoperative visible fluorescence is a predictor for early tumor progression, transformation into more aggressive higher-grade tumors, and shorter survival in LGG patients. Therefore, visible 5-ALA fluorescence is an intraoperative marker of unfavorable prognosis during surgery of LGG.

**Abstract:**

The prediction of the individual prognosis of low-grade glioma (LGG) patients is limited in routine clinical practice. Nowadays, 5-aminolevulinic acid (5-ALA) fluorescence is primarily applied for improved intraoperative visualization of high-grade gliomas. However, visible fluorescence is also observed in rare cases despite LGG histopathology and might be an indicator for aggressive tumor behavior. The aim of this study was thus to investigate the value of intraoperative 5-ALA fluorescence for prognosis in LGG patients. We performed a retrospective analysis of patients with newly diagnosed histopathologically confirmed LGG and preoperative 5-ALA administration at two independent specialized centers. In this cohort, we correlated the visible intraoperative fluorescence status with progression-free survival (PFS), malignant transformation-free survival (MTFS) and overall survival (OS). Altogether, visible fluorescence was detected in 7 (12%) of 59 included patients in focal intratumoral areas. At a mean follow-up time of 5.3 ± 2.9 years, patients with fluorescing LGG had significantly shorter PFS (2.3 ± 0.7 vs. 5.0 ± 0.4 years; *p =* 0.01), MTFS (3.9 ± 0.7 vs. 8.0 ± 0.6 years; *p* = 0.03), and OS (5.4 ± 1.0 vs. 10.3 ± 0.5 years; *p* = 0.01) than non-fluorescing tumors. Our data indicate that visible 5-ALA fluorescence during surgery of pure LGG might be an already intraoperatively available marker of unfavorable patient outcome and thus close imaging follow-up might be considered.

## 1. Introduction

Low-grade gliomas (LGG) are a common tumor entity in neurosurgical practice accounting for approximately 10–20% of all primary brain tumors [1]. In these tumors, a more extensive resection demonstrated to markedly improve patient outcome and thus maximum safe tumor resection is the treatment of choice [2,3,4,5,6]. Generally, the median overall survival time of patients suffering from LGG is approximately 10–15 years [5,6,7,8]. However, the prediction of the individual prognosis of LGG patients is limited in routine clinical practice in certain cases despite the use of molecular markers. In this sense, some patients unexpectedly show early malignant transformation after surgery and rapid tumor progression [9,10].

In the last decades, intraoperative visualization of tumor tissue of high-grade gliomas with the assistance of 5-aminolevulinic acid (5-ALA)-induced fluorescence was increasingly applied in the neurosurgical field [11,12,13]. In a multicenter phase III trial published in 2006, Stummer et al. showed a significantly higher portion of complete resections and prolonged progression-free survival (PFS) of high-grade glioma patients with 5-ALA fluorescence-guided surgery as compared to conventional white-light microscopy [13]. In 2010, we analyzed for the first time the 5-ALA fluorescence behavior in a series of radiologically suspected LGG [14]. According to these first observations, we found visible 5-ALA fluorescence in circumscribed intratumoral areas that histopathologically corresponded to regions of malignant transformation [14]. In a subsequent study from 2013, we demonstrated a significant correlation of visible 5-ALA fluorescence with histopathological criteria of malignancy such as mitotic rate, cell density, nuclear pleomorphism and proliferation rate [15]. Interestingly, we also observed single patients showing visible 5-ALA fluorescence in focal intratumoral areas despite LGG histology in the entire tumor [14,15]. In a preliminary analysis in 2011, we hypothesized that visible 5-ALA fluorescence in a pure LGG might be an indicator for aggressive tumor behavior [16].

Based on these preliminary data, the aim of this study was thus to retrospectively analyze a series of newly diagnosed LGG surgically treated at two independent specialized centers after preoperative 5-ALA administration with adequate follow-up time. To this end, we correlated the visible intraoperative 5-ALA fluorescence status with patient outcome parameters such as PFS, malignant transformation-free survival (MTFS) and overall survival (OS). Moreover, we investigated further prognostic factors such as isocitrate dehydrogenase (IDH) mutational status, pattern of contrast-enhancement (CE) on magnetic resonance imaging (MRI), extent of resection, patient age and histological subtype in the cohort of LGG patients.

## 2. Materials and Methods

### 2.1. Patient Recruitment

In this study, we screened our 5-ALA database for adult patients (≥18 years) receiving tumor resection of an histopathologically confirmed diffusely infiltrating LGG World Health Organization (WHO) grade II after preoperative 5-ALA administration at the Department of Neurosurgery, Medical University Vienna between 2008 and 2017. The retrospective study protocol was approved by the ethics committee of the Medical University of Vienna (reference No. 419/2008, amendment). Additionally, we included patients with histopathologically confirmed diffusely infiltrating LGG (WHO grade II) and preoperative 5-ALA administration derived from a prospective study at the University of California, San Francisco, between 2016 and 2017 (clinical trial registration number NCT01116661) [17]. In the current study, we only included patients with newly diagnosed LGG and thus excluded patients with recurrent tumors.

### 2.2. Preoperative Imaging

Generally, diagnostic magnetic resonance imaging (MRI) including contrast-enhanced images was performed in the preoperative course as well as additional images for integration into neuronavigation. Based on the pattern of CE, an experienced neuroradiologist (JF) classified the type of CE as “no,” “patchy/faint” or “focal” CE as described previously [14,15,17,18]. Furthermore, the tumor localization was categorized in 7 main regions: frontal, parietal, temporal, occipital, central, insular and thalamic. 

### 2.3. Tumor Resection

In all patients, tumor resection was performed under white-light microscopy with assistance of neuronavigation. In order to minimize the risk of a postoperative neurological deterioration, navigation with diffusion tensor imaging (DTI), brain stimulation/mapping, awake surgery and/or intraoperative electrophysiological monitoring were applied in dependence of the tumor localization. In each patient, 5-ALA (20 mg/kg bodyweight) was orally administered approximately 3 h prior to induction of anesthesia. According to the strategy at both institutions, the presence of visible fluorescence (yes or no) and the intratumoral fluorescence pattern (focal or diffuse) was investigated during surgery repeatedly with violet-blue excitation light in different intratumoral areas using a modified neurosurgical microscope (NC4/Pentero, Carl Zeiss Surgical GmbH, Oberkochen, Germany). 

### 2.4. Histopathology

Tumor diagnosis was initially established by the local neuropathology team according to the WHO classification at the time of resection in each patient at both institutions [19,20]. In the present study, only patients with tumor diagnosis of a diffusely infiltrating LGG WHO grade II were included. In cases diagnosed before 2016, diagnoses were updated for this study according to the current diagnostic criteria as specified in the revised 4th edition of the WHO Classification (2016) including determination of the required molecular markers [19]. Midline lesions were investigated for histone H3K27 mutational status using immunohistochemical stainings for H3K27M and H3K27me3. With regard to the IDH mutational status, immunohistochemical staining was performed by applying the IDH1-R132H mutation-specific antibody. In case of negativity, direct sequencing was conducted to detect alternative mutations in IDH1 or IDH2 according to the guidelines of the European Association for Neuro-Oncology (EANO) [21]. Due to the small sample size of IDH-wildtype tumors in our cohort (*n* = 5), comparison of histological subtypes was performed only between IDH-mutant diffuse astrocytomas and IDH-mutant oligodendrogliomas as strictly defined by the WHO Classification (2016).

### 2.5. Postoperative Course

Following surgery after 5-ALA administration, patients were protected from strong light sources for at least 24 h (Medical University Vienna, Wien, Austria) or 72 h (University of California, San Francisco, CA, USA) to avoid potential drug-related phototoxicity. In order to evaluate the extent of tumor resection, a post-operative MRI was generally conducted within 72 h following surgery. Based on postoperative MRI data, a complete resection was defined as tumor removal of ≥99% of FLAIR/T2 alterations.

All patients were discussed in an interdisciplinary tumor board to determine the postoperative management. According to this tumor board recommendations, immediate postoperative adjuvant therapy following surgery was performed in three patients (5%) with large non-resectable tumor remnants. In two of these patients, radiochemotherapy was initiated and in one patient only radiotherapy was conducted due to lack of O-6-methylguanine-DNA methyltransferase (MGMT) gene promoter methylation. In the remaining patients (*n* = 56), no primary postoperative adjuvant therapy after surgery was initiated. 

### 2.6. Follow-Up Period

In the postoperative follow-up period, regular MRI images were performed in time intervals according to both institutions’ practice. A neuroradiological progression was based in this study on the current “Response Assessment in Neuro-Oncology” (RANO) criteria [22]. In this sense, we defined a “tumor progression” as development of new lesions or increase of CE, increase of T2 or FLAIR lesion >25% and tumor-related death [22].

In case of tumor progression/suspected malignant transformation during the follow-up period, patients were discussed in an interdisciplinary neurooncological tumor board and treatment options were determined accordingly including reoperation and/or adjuvant therapy. In case of reoperation, tumor tissue was histopathologically examined for presence of high-grade pathology. A “malignant transformation” was defined as progression of an initial LGG (WHO grade II) to anaplastic glioma (WHO III) or glioblastoma (WHO IV).

Patients with a last clinical examination as well as availability of neuroradiological imaging less than 6 months after tumor resection were classified as lost to follow-up. PFS was defined as time from initial surgery to neuroradiological progression according to the RANO criteria. [22] MTFS was calculated as time from initial surgery to time of reoperation with histological/molecular confirmation of malignant transformation. OS was defined as time from initial surgery to patient’s death.

### 2.7. Statistics

Statistical analysis was performed, and Kaplan–Meier plots created using the statistical software using IBM SPSS Statistics (Version 22.0, Armonk, NY, USA). All data are presented as absolute counts or means ± standard deviation. Patients’ age was categorized to age ≤50 years and >50 years, according to the LGG prognostic scoring system at the University of California, San Francisco [23]. Differences in 5-ALA fluorescence status between dichotomous variables (age >50 years, CE on MRI, extent of resection, histological subtype, IDH status) were compared using chi-square tests. In order to analyze differences in outcome data (PFS, MTFS, OS) for the categorical variables (5-ALA fluorescence status, patient age >50 years, CE on MRI, extent of resection, histological subtype, IDH mutational status) Kaplan–Meier estimators were generated, and log-rank tests were conducted. Differences were considered to be statistically significant at a two-sided significance level *p* < 0.05.

## 3. Results

In the present study, 59 patients with resection of a newly diagnosed, histopathologically confirmed diffusely infiltrating LGG (WHO grade II) after 5-ALA administration at two specialized centers were included. In none of the patients were any severe 5-ALA related adverse reactions observed.

### 3.1. Patient Characteristics

The median age of the study cohort was 38.8 years (range 20.4–65.5 years) with a male to female ratio of 1.3:1. The most common tumor localization was the frontal lobe (*n* = 27; 46%), followed by the central region (*n* = 11; 19%) and the temporal lobe (*n* = 10; 17%). In the majority of LGG, no CE was detected on preoperative MRI (*n* = 43; 73%). In the remaining patients, patchy/faint (*n* = 11; 19%) and focal CE (*n* = 5; 8%) was present. According to the WHO 2016 classification [19], diffuse astrocytoma IDH-mutant was diagnosed in 29 patients (49%), oligodendroglioma IDH-mutant and 1p19q-codeleted in 23 patients (39%), and diffuse astrocytoma IDH-wildtype in 3 patients (5%). In four patients (7%) tumor reclassification according to the updated WHO 2016 classification [19] was not possible due to insufficient sample material. Further details on patient characteristics are provided in Table 1.

### 3.2. Tumor Resection and Intraoperative 5-ALA Fluorescence

During surgery, visible fluorescence was detected in 7 patients (12%). In all of these fluorescing cases, visible fluorescence was observed only in focal intratumoral areas. In contrast, no visible fluorescence was found in any intratumoral part in the majority of patients (*n* = 52; 88%). With regard to the IDH mutational status, IDH wildtype gliomas were significantly more common in tumors with visible 5-ALA (*n* = 2 of 7 fluorescing tumors; 29%) as compared to non-fluorescing tumors (*n* = 3 of 52 non-fluorescing tumors; 6%; *p* = 0.04). However, the presence of visible fluorescence did not correlate with age at diagnosis (*p* = 0.47), the pattern of CE on MRI (*p* = 0.32), extent of resection (*p* = 0.25) and histological subtype (diffuse astrocytoma IDH-mutant vs. oligodendroglioma IDH-mutant; *p* = 0.88).

### 3.3. Postoperative Course and Follow-Up

**Figure 1:**
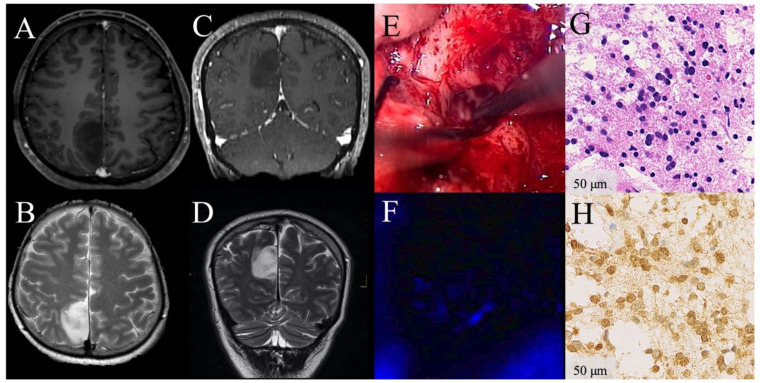
Illustrative clinical course of a patient with absence of visible 5-aminolevulinic acid (5-ALA) florescence during surgery of a low-grade glioma. Preoperative contrast-enhanced (CE) magnetic resonance images demonstrate a radiologically suspected LGG in the parietal lobe with patchy/faint CE on T1-weighted axial (**A**) and coronal images (**C**) and hyperintensity on T2-weighted sequences (**B**,**D**). Under white-light microscopy whitish glioma tissue is shown (**E**). Under violet-blue excitation light, no 5-ALA fluorescence is visible during the entire procedure (**F**). The corresponding histopathological sample from this non-fluorescing area reveals diffusely infiltrating astrocytoma WHO grade II tissue according to the WHO 2016 criteria in the H&E stain (**G**). Immunhistochemical staining for IDH mutation is highly positive (**H**). In the postoperative follow-up, this patient is still alive, more than 11.3 years after initial surgery.

**Figure 2:**
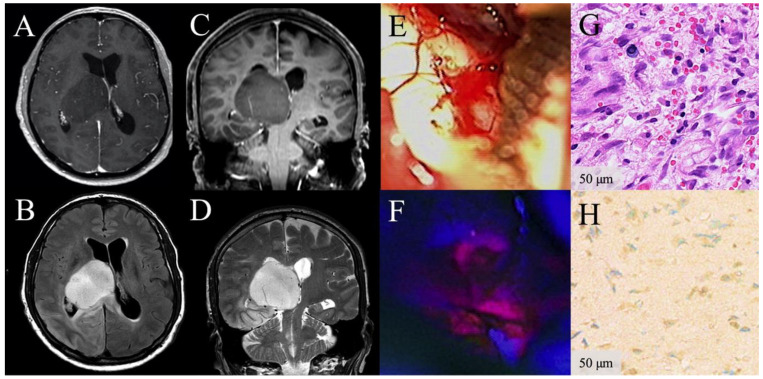
Illustrative clinical course of a patient with focal visible 5-aminolevulinic acid (5-ALA) fluorescence during surgery of a low-grade glioma. Preoperative contrast-enhanced (CE) magnetic resonance images demonstrate a radiologically suspected LGG in the thalamus without CE on T1-weighted axial (**A**) and coronal images (**C**), but hyperintensity on FLAIR (**B**) and T2-weighted sequences (**D**). Under white-light microscopy whitish glioma tissue is present (**E**). Under violet-blue excitation light, the corresponding area shows focal 5-ALA fluorescence (**F**). The corresponding histopathological sample reveals diffusely infiltrating astrocytoma WHO grade II tissue (H3K27 wildtype) according to the WHO 2016 criteria in the H&E stain and no malignant glioma tissue is found despite the visible fluorescence. (**G**). Immunhistochemical staining for IDH mutation (**H**) and sequencing for other IDH1 or IDH2 mutations were negative (IDH wildtype glioma) (**H**). This patient survived only 1.5 years and showed early radiological tumor progression 6 months after surgery.

In our study cohort, a complete resection was achieved in 30 patients (51%) based on postoperative MRI within 72 h following surgery. In the remaining 29 patients (49%) an incomplete resection was present. With regard to the postoperative follow-up, MRI investigations during a time period of at least 6 months were available in 55 patients (93%). Therefore, 4 patients (7%) with a follow-up time interval less than 6 months after surgery had to be excluded from further analyses. In the cohort of 55 patients, the mean follow-up time was 5.3 ± 2.9 years. Illustrative cases of 2 patients with different clinical course in one LGG with visible fluorescence and a further LGG with absence of visible fluorescence are provided in Figure 1 and Figure 2.

#### 3.3.1. Progression Free Survival

**Figure 3:**
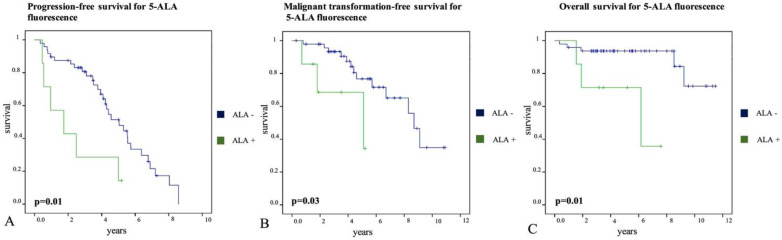
Visible 5-ALA fluorescence and patient prognosis in patients with low-grade gliomas. Kaplan–Meier curves demonstrate survival of patients showing visible intraoperative fluorescence (5-ALA +) compared to patients without any intratumoral fluorescence (5-ALA −). Tumor progression was defined as development of new lesions or increase of contrast-enhancement and/or increase of T2 or FLAIR lesion >25% on MRI or tumor-related death. Malignant transformation was defined as progression to anaplastic glioma (WHO III) or glioblastoma (WHO IV) at reoperation. Overall survival was calculated as time from initial surgery to patient’s death. A significantly shorter progression-free survival (2.3 ± 0.7 vs. 5.0 ± 0.4 years; *p* = 0.01; (**A**)), malignant transformation-free survival (3.9 ± 0.7 vs. 8.0 ± 0.6 years; *p* = 0.03; (**B**)), and overall survival (5.4 ± 1.0 vs. 10.3 ± 0.5 years; *p* = 0.01; (**C**)) was observed in patients with fluorescing LGG during surgery as compared to non-fluorescing tumors.

During the follow-up period, 37 of 55 patients (67%) developed tumor progression on follow-up MRI examinations at a mean interval of 3.8 ± 2.4 years. We found a significantly shorter PFS in patients with fluorescing tumors (2.3 ± 0.7 years, 95%-CI: 1.0–3.7) as compared to gliomas with absence of visible fluorescence (5.0 ± 0.4 years, 95%-CI: 4.2–5.7; *p* = 0.01, see Figure 3A). Moreover, patients with diffuse astrocytoma IDH-mutant had a significantly shorter PFS (3.9 ± 0.5 years, 95%-CI: 2.8–4.9) than patients with oligodendroglioma IDH-mutant (5.8 ± 0.6 years, 95%-CI: 4.7–6.9; *p* = 0.04). In contrast, no significant difference in PFS was found for patient age >50 years (*p* = 0.56), extent of resection (*p* = 0.47), pattern of CE on preoperative MRI (*p* = 0.07), and IDH mutational status (*p* = 0.10). Further details are shown in Table 2.

#### 3.3.2. Malignant Transformation-Free Survival

Furthermore, a second surgery was performed in 21 of 55 patients (38%) during the follow-up period. At the time of second surgery, malignant transformation based on postsurgical histopathological analysis occurred in 16 of 21 patients (76%) at a mean interval of 4.5 ± 2.7 years after primary surgery. In detail, 11 patients showed progression to anaplastic glioma (WHO grade III) and in 5 patients, malignant transformation to glioblastoma (WHO grade IV) was observed. In the remaining 5 patients (24%), histopathological diagnosis again revealed a WHO grade II glioma after second surgery. According to our data, we found a significantly shorter MTFS in patients with fluorescing tumors (3.9 ± 0.7 years, 95%-CI: 2.5–5.4) as compared to gliomas with absence of visible fluorescence (8.0 ± 0.6 years, 95%-CI: 6.9–9.1; *p* = 0.03, see Figure 3B). Moreover, MTFS was significantly shorter in patients with gliomas showing CE on preoperative MRI (patchy/faint or focal CE) at the time of first surgery (6.1 ± 0.7 years, 95%-CI: 4.6–7.5) as compared to gliomas without preoperative CE (none; 8.6 ± 0.7 years, 95%-CI: 7.3–10.0; *p* = 0.048). However, there was no difference in MTFS for patient age >50 years (*p* = 0.2), extent of resection (*p* = 0.07), IDH mutational status (*p* = 0.60) and histological subtype (*p* = 0.57). Further details are provided in Table 3.

#### 3.3.3. Overall Survival

During the follow-up period, 8 patients (15%) of our study cohort died at a mean interval of 3.8 ± 3.6 years due to tumor progression. We found a significantly shorter OS in patients with fluorescing tumors (5.4 ± 1.0 years, 95%-CI: 3.5–7.3) as compared to gliomas with absence of visible fluorescence (10.3 ± 0.5 years, 95%-CI: 9.3–11.2; *p* = 0.01, see Figure 3C). Furthermore, OS was significantly shorter in patients with IDH wildtype gliomas (2.3 ± 0.3 years, 95%-CI: 1.7–3.0) as compared to IDH mutant gliomas (10.1 ± 0.5 years, 95%-CI: 9.1–11.1; *p* = 0.003). Moreover, patients with complete tumor resection showed a significantly longer OS (10.9 ± 0.4 years, 95%-CI: 10.2–11.6) as compared to patients with incomplete tumor removal (8.6 ± 0.8 years, 95%-CI: 6.9–10.2; *p* = 0.03). However, there was no difference in OS for patient age >50 years (*p* = 0.16), pattern of CE on preoperative MRI (*p = 0.13*), and histological subtype (*p* = 0.53). Further details are shown in Table 4.

## 4. Discussion

In previous studies, we were able to demonstrate that visible 5-ALA fluorescence during surgery of radiologically suspected LGG represents a marker for malignant transformation [14,15,17]. According to our observations, however, focal 5-ALA fluorescence might also occur during surgery despite pure LGG histopathology in the entire tumor. Already in 2011, we hypothesized that visible 5-ALA fluorescence might be an indicator for aggressive tumor behavior in pure LGG based on our preliminary data [16]. This is the first study investigating the value of 5-ALA fluorescence in pure LGG in two specialized independent centers with a sufficiently long follow-up time for newly diagnosed LGG.

### 4.1. Present Study

We therefore designed the current study and investigated if visible 5-ALA fluorescence represents an intraoperative prognostic marker in pure LGG. To this end, we analyzed a cohort of patients with surgically resected LGG and preoperative 5-ALA administration at two specialized centers. In this study cohort, we correlated visible 5-ALA fluorescence with specific patient outcome parameters. Additionally, we investigated further prognostic factors such as IDH mutational status, pattern of CE on MRI, extent of resection, patient age and histological subtype in the LGG study cohort. Altogether, we included 59 patients in the current study with a mean follow-up time of 5.3 ± 2.9 years.

#### 4.1.1. 5-ALA Fluorescence and Patient Prognosis

Of the 59 patients, 12% patients demonstrated visible fluorescence in focal intratumoral areas despite pure LGG histology. These findings are in line with previous studies using 5-ALA in LGG showing visible fluorescence in up to 8–22% of cases [15,24,25,26]. It is of note that visible fluorescence was observed only in focal intratumoral areas in all our cases, which is in line with our previous finding in gliomas with non-significant CE [15]. Therefore, 5-ALA guided resection will not improve the extent of resection in these cases. However, others reported visible fluorescence in LGG not only in focal areas, but also diffusely in the whole tumor [26,27]. In this sense, Goryaynov et al. described a diffuse fluorescence pattern in 5 of 9 patients with fluorescing WHO grade II gliomas and in 2 of 5 fluorescing WHO grade I tumors [27]. The reason for these different fluorescence patterns (focal vs. diffuse) is not clear so far. Since 5-ALA itself is non-fluorescent, tumor visualization is dependent on metabolization to Protoporphyrin IX by enzymes of the heme biosynthesis pathway. Recent studies demonstrated that the heme biosynthesis mRNA expression is a promising marker for prognosis in patients WHO Grade II and III gliomas [28,29]. In this sense, the prognostic impact of heme biosynthesis regulation may be reflected in the presence of intraoperative 5-ALA induced fluorescence in pure LGG. Furthermore, it was shown that focal intratumoral areas of 5-ALA fluorescence correlate well with histopathological criteria of malignancy [14,15]. Therefore, we hypothesize that focal areas of visible fluorescence in LGG might represent intratumoral areas of increased biological tumor aggressiveness, which are not yet histopathologically detectable.

According to our data, intraoperative 5-ALA fluorescence in LGG was significantly associated with shorter PFS (mean PFS: 2.3 ± 0.7 years vs. 5.0 ± 0.4 years), MTFS (mean MTFS: 3.9 ± 0.7 years vs. 8.0 ± 0.6 years) and OS (5.4 ± 1.0 years vs. 10.3 ± 0.5 years) compared to patients without fluorescing LGG. In a comparable retrospective study, Jaber et al. found a higher portion (21.6%) of visible 5-ALA fluorescence in a cohort of 74 LGG patients with a median follow-up time of 3.9 years [26]. In this study, the authors found similar results, i.e., a significant correlation of visible 5-ALA fluorescence with PFS (median PFS 0.8 years vs. 3.8 years), MTFS (median MTFS: 3.6 years vs. 5.4 years) and OS (median OS: 4.3 years vs. 5.7 years) in univariate analysis [26]. Therefore, we were able to confirm our preliminary observations [16] as well as the findings of the recent study conducted by Jaber et al. [26] in our current study with a markedly longer follow up time as well as a cohort including patients from two independent specialized centers. Altogether, these studies highlight the value of visible 5-ALA fluorescence for intraoperative identification of patients with aggressive clinical course and poor patient prognosis despite LGG histopathology.

#### 4.1.2. Analysis of Further Prognostic Factors for Patients with LGG

In the current study, we also correlated the 5-ALA fluorescence in LGG with further prognostic factors such as IDH mutational status, pattern of CE on MRI, extent of resection, patient age and histological subtype. Additionally, we analyzed the impact of these known prognostic factors for patient outcome in this patient series of pure LGG.

#### 4.1.3. IDH Mutational Status

The IDH mutational status is an important prognostic molecular marker in the management of patients with LGG. Such IDH mutations are frequently observed in LGG and occur early in tumorgenesis [30]. These mutations are significantly associated with favorable prognosis in LGG. [31,32] Most of the LGG in the present study population were IDH mutated (92%). In contrast to the study of Jaber et al. [26] visible 5-ALA fluorescence was significantly more commonly in IDH wildtype gliomas in our study, suggesting 5-ALA fluorescence as an early predictor of the individual molecular profile already during surgical resection. In accordance with the current literature [33,34], IDH mutation was significantly associated with longer OS in our study cohort of pure LGG. However, we did not find a significant relation between the IDH mutational status and PFS/MTFS. This might be explained by the high heterogeneity in the survival data of IDH wildtype LGG observed in previous studies, suggesting that the IDH mutational status alone might not predict the risk of malignant transformation in the individual patient [35]. Interestingly, it was previously shown that greater extent of resection in IDH wildtype gliomas was associated with improved MTFS and OS [36]. Since 5-ALA fluorescence was observed more common in IDH wildtype tumors, future studies should evaluate if a more aggressive tumor resection in focally fluorescing LGG might improve overall outcome.

#### 4.1.4. CE on MRI, Extent of Resection, Histological Subtype

In this study, CE on preoperative MRI (patchy/faint or focal CE) was present in 27% of LGG, which is in accordance with the reported presence of CE of LGG in literature ranging from 16–50% [17,18,26,37]. In our present study, we did not find a correlation of 5-ALA fluorescence with CE on MRI in our LGG patients. In the literature, Castet et al. reported CE on MRI as an independent negative prognostic factor for OS in pure LGG, especially in IDH mutant tumors [37]. In contrast, Pallud et al. found no association of CE (any pattern) and OS in LGG, but identified nodular CE and CE progression over time as a poor prognostic marker for OS [18]. In our study, the presence of CE on preoperative MRI (patchy/faint or focal CE) was associated with shorter MTFS, suggesting that the increase in blood-brain barrier permeability represents an early sign of focal malignant transformation [38].

There is strong evidence that complete resection of LGG is crucial to improve OS [5,39,40]. In accordance, we found a significantly higher OS in LGG with complete resection (mean OS: 10.9 ± 0.4 years) as compared to incomplete tumor removal (mean OS: 8.6 ± 0.8 years). Additionally, Berger et al. reported that complete resection of LGG results in reduced risk of malignant transformation [41]. Further studies confirmed these initial observations and showed that complete resection was associated with less malignant transformation and tumor progression [40,42]. In the present study, we also found a longer MTFS in patients with complete resection (mean MTFS: 8.8 ± 0.8 years) as compared to incomplete tumor removal (mean MTFS: 6.6 ± 0.6 years). Nevertheless, this difference did not reach statistical significance (*p* = 0.07). In our view, this is probably caused by the small sample size of included patients (*n* = 59) and thus larger studies in LGG patients with a longer follow-up time are needed to answer this specific research question.

Although the histological subtype and patients’ age are well accepted prognostic factors in patients suffering from LGG [7], we were not able to show statistically significant differences in these investigated parameters, except for shorter PFS in diffuse astrocytoma IDH-mutant compared to oligodenroglioma IDH-mutant. Again, we assume that this unexpected fact might be explained by the low number of patients included in our study. With regard to 5-ALA fluorescence, neither extent of resection, histological subtype nor patients’ age did correlate with 5-ALA fluorescence, suggesting that 5-ALA is an independent prognostic factor in patients with LGG.

### 4.2. Clinical Relevance and Future Direction

Since intraoperative focal 5-ALA fluorescence in LGG significantly correlated with patient prognosis in our study and the literature [16,26], we recommend at least close postoperative MRI follow-up to detect tumor progression and malignant transformation at the earliest possible time point. Alternatively, early initiation of adjuvant postoperative therapy might be considered in LGG with focal 5-ALA fluorescence especially in cases where only incomplete tumor resection could be achieved and/or in LGG with an unfavorable molecular tumor profile. As the exact pathophysiological mechanism of 5-ALA fluorescence in LGG with regard to patient prognosis is unclear so far, further detailed studies should investigate fluorescing and non-fluorescing LGG on histopathalogical, immunhistochemical and molecular level.

### 4.3. Limitations

The following limitations of this study have to be considered: (1) First of all, this study had a retrospective design with its known limitations. (2) Moreover, the sample size of our study cohort is relatively low (*n* = 59 patients). However, we already included a second independent specialized center in order to increase the number of patients. Furthermore, our current study cohort is among the largest reported in the current literature. (3) Due to the low number of patients and small subgroups, statistical power of a multivariate survival analysis would have been too low to produce meaningful results and was therefore not undertaken. (4) There is a possibility that some of the five IDH-wildtype tumors would be assigned a grade 4 according to the upcoming 5th edition of the *Classification of Tumours of the Central Nervous System,* based on the status of additional molecular alterations which are not available in our cohort.

## 5. Conclusions

In the present study, we investigated the value of visible 5-ALA fluorescence in a series of surgically treated LGG at two independent specialized centers for patient prognosis. According to our data, we found a significantly shorter PFS, MTFS and OS in patients with fluorescing LGG as compared to non-fluorescing tumors. Additionally, visible 5-ALA fluorescence was significantly more common in IDH wildtype LGG as compared to IDH mutated tumors. The data of our study thus indicate that visible 5-ALA fluorescence during surgery of pure LGG might be an early marker of unfavorable patient outcome. Consequently, close imaging follow-up might be considered in these patients. Furthermore, future studies should clarify if patients might benefit also from early initiation of adjuvant postoperative therapy in case of focal 5-ALA fluorescence in pure LGG.

## Figures and Tables

**Table 1:** Patients’ characteristics.

		*n*	%
**Number of patients**		59	(100)
Gender			
	male	33	(56)
	female	26	(44)
Tumor localization			
	frontal	27	(46)
	central region	11	(19)
	temporal	10	(17)
	insular	8	(13)
	parietal	2	(3)
	thalamus	1	(2)
Tumor side			
	right	27	(46)
	left	31	(52)
	bilateral	1	(2)
Pattern of contrast-enhancement			
	no	43	(73)
	patchy/faint	11	(19)
	focal	5	(8)
Extent of resection			
	complete	29	(49)
	incomplete	30	(51)
Intraoperative fluorescence			
	yes	7	(12)
	no	52	(88)
Pattern of fluorescence			
	focal	7	(100)
	diffuse	0	(0)
Neuropathology			
Diagnosis (WHO 2016)			
	diffuse astrocytoma, IDH-mutant	29	(49)
	diffuse astrocytoma, IDH-wildtype	3	(5)
	oligodendroglioma, IDH-mutant and 1p19q-codeleted	23	(39)
	diffuse glioma, NOS	4	(7)
IDH mutational status			
	mutant	54	(91.5)
	wildtype	5	(8.5)

IDH = Isocitrate dehydrogenase, NOS = not otherwise specified, 5-ALA = 5-aminolevulinic acid.

**Table 2:** Progression-free survival.

		PFS (Years)	95%-CI (Years)	*p*-Value
Patient age				
	>50 years	5.4 ± 1.2	3.0–7.7	
	≤50 years	4.6 ± 0.4	3.8–5.3	*p* = 0.56
Pattern of CE on MRI				
	patchy/faint, focal	3.9 ± 0.5	2.8–4.9	
	none	5.0 ± 0.5	4.1–6.0	*p* = 0.07
5-ALA fluorescence				
	yes	2.3 ± 0.7	1.0–3.7	
	no	5.0 ± 0.4	4.2–5.7	***p* = 0.01**
Extent of resection				
	complete	5.0 ± 0.5	4.1–5.9	
	incomplete	4.3 ± 0.6	3.2–5.4	*p* = 0.47
Histological subtype				
	oligodendroglioma IDH-mutant	5.8 ± 0.6	4.7–6.9	
	diffuse astrocytoma IDH-mutant	3.9 ± 0.5	2.8–4.9	***p*** **= 0.04**
IDH mutational status				
	wildtype	1.9 ± 0.6	0.7–3.0	
	mutant	4.8 ± 0.4	4.0–5.5	*p* = 0.10

Bold: highlight significant values.

**Table 3:** Malignant transformation-free survival.

		MTFS (Years)	95%-CI (Years)	*p*-Value
Patient age				
	>50 years	9.2 ± 0.3	8.5–9.8	
	≤50 years	7.3 ± 0.6	6.1–8.6	*p* = 0.2
Pattern of CE on MRI				
	patchy/faint, focal	6.1 ± 0.7	4.6–7.5	
	none	8.6 ± 0.7	7.3–10.0	***p*** **= 0.048**
5-ALA fluorescence				
	yes	3.9 ± 0.7	2.5–5.4	
	no	8.0 ± 0.6	6.9–9.1	***p* = 0.03**
Extent of resection				
	complete	8.8 ± 0.8	7.2–10.4	
	incomplete	6.6 ± 0.6	5.4–7.8	*p* = 0.07
Histological subtype				
	oligodendroglioma IDH-mutant	7.5 ± 0.6	6.3–8.7	
	diffuse astrocytoma IDH-mutant	7.3 ± 0.9	5.5–9.1	*p* = 0.57
IDH mutational status				
	wildtype	n.a.	n.a.	
	mutant	n.a.	n.a.	*p* = 0.60

Bold: highlight significant values.

**Table 4:** Overall survival.

		OS (Years)	95%-CI (Years)	*p*-Value
Patient age				
	>50 years	7.7 ± 1.1	5.6–9.8	
	≤50 years	10.2 ± 0.5	9.2–11.2	*p* = 0.16
Pattern of CE on MRI				
	patchy/faint, focal	11.0 ± 0.5	10.0–11.9	
	none	8.9 ± 0.6	7.6–10.1	*p* = 0.13
5-ALA fluorescence				
	yes	5.4 ± 1.0	3.5–7.3	
	no	10.3 ± 0.5	9.3–11.2	***p* = 0.01**
Extent of resection				
	complete	10.9 ± 0.4	10.2–11.6	
	incomplete	8.6 ± 0.8	6.9–10.2	***p* = 0.03**
Histological subtype				
	oligodendroglioma IDH-mutant	10.2 ± 0.7	8.9–11.5	
	diffuse astrocytoma IDH-mutant	9.7 ± 0.7	8.2–11.1	*p* = 0.53
IDH mutational status				
	wildtype	2.3 ± 0.3	1.7–3.0	
	mutant	10.1 ± 0.5	9.1–11.1	***p* = 0.003**

Bold: highlight significant values.

## Data Availability

Data is contained within the article.
